# Safety of Intravenous Application of Mistletoe (*Viscum album* L.) Preparations in Oncology: An Observational Study

**DOI:** 10.1155/2014/236310

**Published:** 2014-05-15

**Authors:** Megan L. Steele, Jan Axtner, Antje Happe, Matthias Kröz, Harald Matthes, Friedemann Schad

**Affiliations:** ^1^Research Institute Havelhoehe, 14089 Berlin, Germany; ^2^Hospital Havelhoehe, 14089 Berlin, Germany; ^3^Institute for Social Medicine, Epidemiology and Health Economics, Charité-University Medical Centre, 10117 Berlin, Germany; ^4^Institute of Integrative Medicine, Witten/Herdecke University, 58313 Herdecke, Germany

## Abstract

*Background*. Traditional mistletoe therapy in cancer patients involves subcutaneous applications of *Viscum album* L. preparations, with doses slowly increasing based on patient responses. Intravenous infusion of high doses may improve therapeutic outcomes and is becoming more common. Little is known about the safety of this “off-label” application of mistletoe. *Methods*. An observational study was performed within the Network Oncology. Treatment with intravenous mistletoe applications is described. The frequency of adverse drug reactions (ADRs) to intravenous mistletoe applications was calculated and compared to ADR data from a study on subcutaneous applications. *Results*. Of 475 cancer patients who received intravenous infusions of Helixor, Abnoba viscum, or Iscador mistletoe preparations, 22 patients (4.6%) reported 32 ADRs of mild (59.4%) or moderate severity (40.6%). No serious ADRs occurred. ADRs were more frequently reported to i.v. mistletoe administered alone (4.3%), versus prior to chemotherapy (1.6%). ADR frequency differed with respect to preparation type, with Iscador preparations showing a higher relative frequency, compared to Abnoba viscum and Helixor. Overall, patients were almost two times less likely to experience an ADR to intravenous compared to subcutaneous application of mistletoe. *Conclusion*. Intravenous mistletoe therapy was found to be safe and prospective studies for efficacy are recommended.

## 1. Introduction

European mistletoe (*Viscum album* L.) extracts are amongst the most frequently prescribed complementary therapies for cancer patients in Europe, especially in Germany, Switzerland, and Austria [1]. Anthroposophic mistletoe preparations (AbnobaVISCUM, Helixor, Iscador, Iscucin) are most commonly applied via subcutaneous (s.c.) injection starting at a low dose, which is slowly increased over time based on individual patient responses. Increasingly mistletoe preparations are also applied at high initial doses, either by s.c. injection, as an intravenous (i.v.) infusion, or intratumoural (i.t.), depending on the location of the tumour, disease stage, and experience of the physician [2–5]. There appears to be building evidence for safety, improvement of quality of life, and reduction in adverse drug reactions (ADRs) to conventional therapies associated with traditional s.c. mistletoe therapy. It is suggested, however, that high dose i.v. mistletoe infusions might be even more effective and might also reduce the risk of tumour metastasis or recurrence, possibly through modulation of the immune system and antiangiogenesis effects [6–10]. In a study assessing humoral cell responses in cancer patients' serum to mistletoe extracts administered by s.c. compared to i.v. application, a distinctly greater stimulant effect on immune cell populations and granulocyte-colony stimulating factor production was observed in i.v. patients [11]. Therefore, the focus of the current study is on the i.v. administration of mistletoe preparations.

Compared to s.c. mistletoe therapy, there have been relatively few published studies investigating the clinical benefits or safety of i.v. administration of mistletoe preparations. Improvements in well-being and quality of life are the most commonly reported, particularly in frail patients with advanced or end-stage cancer, or beneficial effects regarding cancer-related low mood, fatigue, and pain [3, 6, 12–16]. In clinical practice, i.v. mistletoe therapy is often given concomitantly to conventional chemotherapy [17]. In a phase II study by Büssing et al., i.v. administration of mistletoe preparations (Iscador Mali special) showed significant beneficial effects with respect to chemotherapy-related adverse events. I.v. application of Iscador Mali special at concentrations between 1 and 5 mg was shown to be safe and tolerable [18]. Perioperative i.v. applications of mistletoe preparations (Iscador Mali and Quercus) have been shown to significantly minimise the immune suppression triggered by anaesthesia and operation stress [19–21]. We also found a prospective, randomised, controlled trial in which colorectal cancer patients seemed to benefit in terms of survival from combined postoperative chemotherapy and i.v. mistletoe therapy (Isorel) compared to chemotherapy alone [22]. However, the methodological quality of this and other studies assessing the safety and effectiveness of mistletoe therapy has been criticised [1]. In addition, other studies have involved patients receiving mistletoe preparations via different combinations of s.c., i.v., and i.t. administration but did not assess separately the efficacy or safety of the specific forms of administration.

Although increasingly used in clinical practice, i.v. application of anthroposophic mistletoe preparations is still classified as “off-label” use. While s.c. mistletoe therapy has proven to be well-tolerated [23–25], i.v. mistletoe therapy often involves much higher doses, and limited research has been conducted regarding the safety of this application [19, 20]. The present study describes the use of i.v. mistletoe therapy under standard clinical practice within an integrative oncological setting in Germany [26]. In addition to summarising patient demographics and the mistletoe preparations and doses received, the frequency and severity of i.v. mistletoe-related ADRs are assessed and compared to results from our previous study on the safety of s.c. mistletoe [27].

## 2. Methods

### 2.1. Study Design and Data Sources

A multicentre, observational study was carried out within the Network Oncology (NO), a conjoint clinical registry of German hospitals and out-patient practitioners specialised in anthroposophic medicine [26, 28]. As described previously, documentation officers extract patient information, cancer diagnoses, therapies, adverse events, and disease progress from patient files and record data using the QuaDoSta (Quality management, Documentation, and Statistics) software that was developed at Havelhoehe Research Institute [26, 29]. Detailed information about mistletoe therapies received, including dosage data, therapy start and end dates, and related adverse events, is documented. The NO was designed to be compliant with the European Directive on Data Protection, which has implications for record linkage studies, databases, and registers, and the NO-project has received a positive vote from the ethical committee of the Medical Association Berlin [26, 30]. For the present study, data from the medical records of all consenting patients treated between 2003 and 2013 were assessed. Data were recorded in the QuaDoSta between 2010 and 2013 and analysed in 2013. All analyses were conducted and figures were created with R version 2.15.1 [31].

### 2.2. Selection and Analysis of Patient Data

All patients with a valid identification number, birth date, gender, cancer diagnosis date, ICD-10 code, and at least a start or end date for i.v. mistletoe therapy were included in the final analyses. Descriptive statistics were used to describe patient demographics and the disease stage of patients at diagnosis was described according to Union for International Cancer Control (UICC) staging. The Wilcoxon rank sum (*W*) test was used to test for differences between groups. Types of mistletoe and conventional therapies received are summarised.

### 2.3. Analysis of Safety Data

All mistletoe-related adverse events reported by physicians were assessed by the investigators. While increases in body temperatures (especially up to 38°C) are common and often described as a desired or expected effect, an increase in body temperatures >38°C (pyrexia) is internationally regarded as an ADR [32]. In addition, i.v. mistletoe infusions are sometimes accompanied by local reactions surrounding old s.c. application sites. When <5 cm in diameter, these events are considered as expected effects, but when >5 cm, they are recorded as ADRs. Therefore, increased body temperatures >38°C and local reactions >5 cm, along with all other adverse events, were considered as suspected mistletoe ADRs if a causal relationship between i.v. applied mistletoe and an event was described by physicians as at least a reasonable possibility. Decisions of causality were made based on consideration of the dates of mistletoe infusion, of the adverse event, and of other therapies received and on therapy types and doses received, and whether reactions made sense based on known pharmacological activity of therapies and on patient history. I.v. mistletoe is often given on the same day as chemotherapy meaning there is no way to confidently distinguish the causality of some ADRs. However, if given on the same day, mistletoe is almost always administered as the first infusion, before chemotherapeutic premedication (e.g., dexamethasone, prednisolone) and subsequent chemotherapeutic drugs; thus, acute ADRs can be attributed to mistletoe in these cases. All suspected ADRs were classified as MedDRA 15.0 preferred terms (developed under the auspices of the International Conference on Harmonization (ICH)) and grouped by System Organ Class (SOC) [32, 33]. ADRs were evaluated in terms of severity according to the Common Terminology Criteria for Adverse Events (CTCAE) v4.0 [34] and designated as serious or nonserious according to ICH guidelines [32]. Numbers of ADRs per person were determined and the management and outcome of ADRs are summarised. In order to examine whether experiencing an ADR might be dose dependent, the doses at which patients experienced ADRs were considered in relation to the doses to which patients were exposed. Basic information about patients treated with i.v. mistletoe and the relative frequency of experiencing an ADR to i.v. mistletoe therapy was compared to results from a previous study on the safety of s.c. mistletoe therapy [27].

## 3. Results

### 3.1. Patient and Treatment Characteristics

**Figure 1:**
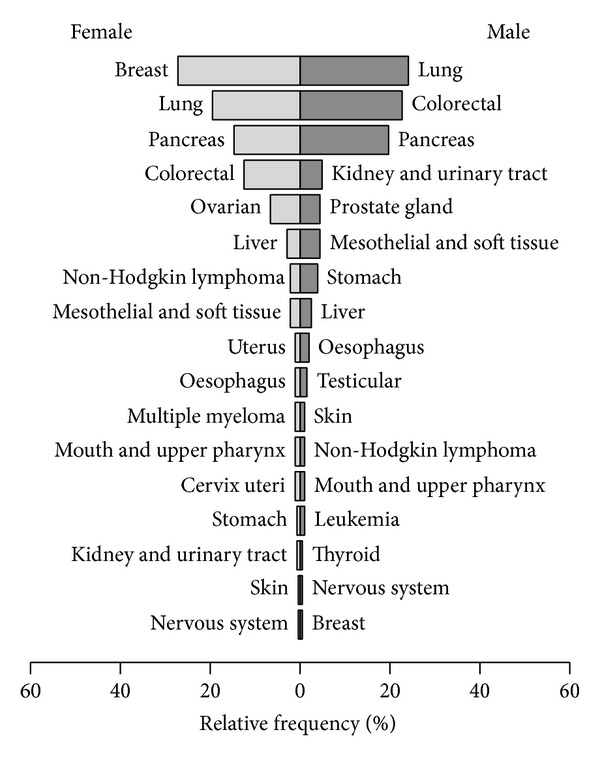
Gender-specific percentages of tumour entities in patients treated with mistletoe preparations via intravenous application.

From a total of 4695 cancer patients treated between July 2003 and June 2013 that were recorded in the NO database, 2805 patients (62.1%) received mistletoe therapy (all forms of administration) and 478 patients (10.2% of all cancer patients and 16.4% of mistletoe patients) received mistletoe via i.v. infusion at least once. Three i.v. mistletoe patients were excluded due to missing information regarding mistletoe preparation types, leaving 475 patients that were analysed in the current study. The patients consisted of 203 males (42.7%) ranging in age from 23 to 86 years and 272 females (57.3%) ranging from 25 to 91 years. The median age of males was 4 years older than that of females (median = 66 : 62, *W* = 23945, *P* = 0.01329) and the overall median age was 63 years. Lung cancer (23% of all patients) was the most frequent cancer disease treated with i.v. mistletoe infusions, followed by pancreatic (18%), colorectal (17%), and breast (17%) cancers (Figure 1). At the time of diagnosis, 46 patients (10%) had a UICC stage of I, 77 (16%) were stage II, 106 (22%) were stage III, and 162 (34%) were stage IV. The UICC stage at diagnosis was not known or applicable for 84 (18%) patients. I.v. mistletoe therapy commenced between 1 day and 16 years (median = 4.3 months) after first diagnosis. At the commencement of i.v. mistletoe therapy, UICC stage was documented for 70% of patients. Of these, 7% were stage II, 18% were stage III, and 75% were stage IV.

**Figure 2:**
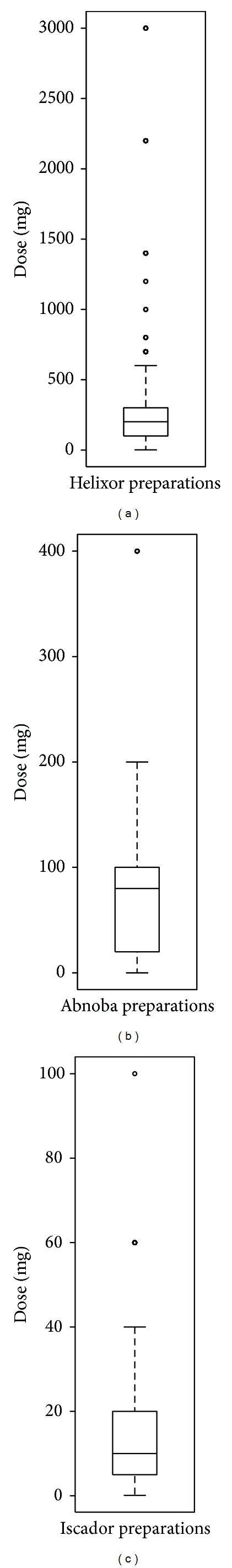
Doses at which intravenous mistletoe applications were given with respect to preparation type. Horizontal lines within boxes show median values, boxes show 25–75% data ranges, the whiskers extend by 1.5x the interquartile range, and circles represent outliers. Note the large differences in scale of the *y*-axes.

Like conventional cancer therapies, mistletoe therapy varies based on cancer type and stage, patient history (and preference), and physician judgement. Application frequencies, durations of therapy, and whether other non-i.v. applications of mistletoe were received are summarised in Table 1. I.v. mistletoe patients mostly received Helixor Mali preparations, followed by Abnoba viscum Fraxini and Helixor Abietis (Table 2). Due to differences in their production, mistletoe dose ranges vary significantly depending on the preparation type. Administered doses of Helixor preparations ranged from 1 mg to 3000 mg with a median dose of 200 mg. Abnoba viscum doses ranged from 0.02 mg to 400 mg with a median of 80 mg and Iscador doses ranged from 0.1 mg to 100 mg with a median of 10 mg (Figure 2).

In addition to mistletoe therapy, 81.5% of patients received a systemic therapy (chemotherapy = 77.5%, immunotherapy = 14.3%, hormonal therapy = 13.1%, bisphosphonates = 11.6% and signal transduction inhibitors = 6.3%), 78.5% of patients had surgery, and 34.1% received radiation therapy. While 445 patients received i.v. mistletoe exclusively (not in combination with another therapy type) at least once, the numbers of patients who received different combinations of therapies within close temporal proximity (very often on the same day) are shown in Table 3. In the cases where i.v. mistletoe was administered alongside chemotherapy (201 from 368 patients who had chemotherapy: 55%), it was documented that 63% received corticosteroids such as prednisolone or dexamethasone as antiemetic or antiallergic medication at least once after administration of i.v. mistletoe and before chemotherapy.

### 3.2. Adverse Drug Reactions Attributed to Intravenous Mistletoe Therapy

Of 475 i.v. mistletoe treated patients, 22 patients (4.6%) experienced a total of 32 ADRs (Table 4). Fifteen patients had one ADR, four had two ADRs, and three had three ADRs. The most frequent ADR was pyrexia (1.7% of all patients), followed by pruritus (1.3%) and urticaria (0.6%). In terms of System Organ Class, most ADRs were “general disorders and administration site conditions” (46.9% of ADRs, 2.1% of total patients) or “skin and subcutaneous tissue disorders” (31.2% of ADRs, 2.1% of total patients). The two ADRs described as “local reactions” refer to redness and tenderness with diameters of >5 cm surrounding old s.c. application sites. All ADRs were classified as either mild (59.4%) or moderate (40.6%) and no serious ADRs occurred.

In terms of ADR management, one patient had a pause in therapy, two patients had a reduction in mistletoe dose, the mistletoe preparation was changed for six patients, and therapy was stopped in six patients. One patient with a localised rash was given Tavegil (antihistamine) and Combudoron gel (anthroposophic remedy) was applied. Two patients were treated for pruritus: one received a Fenistil tablet (antihistamine) and Calcium Quercus (anthroposophic remedy) and the other received i.v. Fenistil. A patient with myalgia (muscle pain) was treated with Ibuprofen. All patients completely recovered from i.v. mistletoe-related ADRs without sequelae.

### 3.3. Investigation of Patients Who Experienced Adverse Drug Reactions to Intravenous Mistletoe Therapy

Of the 22 patients who experienced ADRs, six patients had cancer of the pancreas, three of the breast, two of the colon, two of the rectum, two of the stomach, two of the lung, two of the ovary, one of the uterus, one of the testis, and one patient had non-Hodgkin lymphoma. Of 445 patients who were exposed to i.v. mistletoe therapy alone, 19 (4.3%) patients experienced an ADR. In comparison, only 3 (1.6%) from 187 patients who received i.v. mistletoe prior to chemotherapy were recorded to have experienced an ADR. Of these, one patient experienced pyrexia (38.5°C) following i.v. mistletoe (Helixor Mali), dexamethasone, and chemotherapy with fluorouracil, docetaxel, folinic acid, and oxaliplatin. One week later the patient had the same treatment minus the mistletoe and reacted in the same way; therefore, it is possible that the initial reaction was also caused by the chemotherapy only and not mistletoe. The second patient experienced pyrexia (40°C) after i.v. mistletoe (Helixor Mali) and gemcitabine (no corticosteroids), while details regarding prechemotherapeutic medications were not available for the third patient who experienced pyrexia (39.5°C) after i.v. mistletoe (Iscador Mali) and doxorubicin.

For ten out of the 22 patients that experienced an ADR to i.v. mistletoe, it was their first i.v. mistletoe application, and for three of those, their first ever exposure to mistletoe. Eight patients had an ADR after a significant dose increase, one patient had an ADR after a change in preparation, and for three patients there is no plausible explanation. Twelve patients continued i.v. mistletoe therapy with no additional ADRs, seven patients had no further i.v. applications, and three patients had one or more ADRs to further i.v. applications.

In order to investigate whether administration of high doses is associated with an increased risk of experiencing an ADR, doses at which ADRs occurred must be assessed in relation to the doses to which patients were exposed (Figure 2). ADRs to Helixor extracts occurred in response to doses ranging from 100 mg to 400 mg with a median ADR dose of 150 mg (median exposure dose was 200 mg). For Abnoba viscum, ADR doses ranged from 10 mg to 400 mg with a median of 20 mg (median exposure dose was 80 mg) and for Iscador, from 5 mg to 20 mg with a median of 12.5 mg. Therefore, only for Iscador was the median ADR dose (12.5 mg) higher than the median exposure dose (10 mg). Overall, exposure to high doses of mistletoe did not appear to increase the frequency of ADRs.

The absolute and relative frequencies of patients experiencing an ADR and the types of ADRs with respect to preparation type are shown in Table 2. Iscador Mali special, Abnoba viscum Quercus, Iscador Mali, and Iscador Quercus show the highest relative frequencies of all preparation types. The numbers of patients exposed to different preparation types vary largely, however, making it difficult to draw strong conclusions regarding associations of increased ADR risk with certain preparation types.

### 3.4. Comparison of Intravenous Mistletoe Data to Subcutaneous Mistletoe Data

To assess the safety of i.v. administration of mistletoe extracts in relation to s.c. administration, we compared the present data with results from a previous study on the safety of s.c. mistletoe [27]. There was no significant difference between the ages of patients treated with i.v. or s.c. mistletoe (*W* = 478033, *P* = 0.115). The proportion of female patients who received i.v. mistletoe applications was lower compared to s.c. mistletoe (*W* = 403553, *P* < 0.001), while a greater proportion of i.v. patients had stage IV cancer at diagnosis compared to s.c. patients (*W* = 367656.5, *P* < 0.001). In terms of ADRs, i.v. mistletoe applications resulted in a lower frequency of ADRs (4.6% of exposed patients) compared to s.c. mistletoe applications (8.4% of exposed patients) (OR = 0.53, CI = 0.33–0.82, *P* = 0.005) [27].

## 4. Discussion

The present study summarised the use of i.v. mistletoe applications in 475 cancer patients and assessed suspected i.v. mistletoe ADRs. Most patients were exposed to preparations of Helixor Mali, followed by Abnoba viscum Fraxini and Helixor Abietis. Helixor Mali preparations (extracts from mistletoe grown on apple trees) are recommended for tumours of the gastrointestinal tract, lower abdomen, and breast, while Abietis preparations (extracts from mistletoe grown on fir trees) are recommended for lung, prostate, and head and neck cancers [35]. Considering that tumours of the lung, breast, colorectal, and pancreas were the most frequent cancer entities, a high frequency of exposure to Mali and Abietis preparations was expected. Furthermore, the majority of patients were UICC stage IV cancer patients at the commencement of i.v. mistletoe therapy, indicating metastatic disease. According to product information, mistletoe preparations with high concentrations of lectins and viscotoxins (e.g., Abnoba viscum Fraxini) are recommended for treatment of metastatic tumour disease [36].

Only 4.6% of all patients that received i.v. mistletoe applications experienced an ADR. All ADRs were either mild or moderate in severity and most patients only experienced one ADR. “General disorders and administration site conditions” (especially pyrexia) were the most common types of ADRs, occurring in 2.1% of patients. “Skin and subcutaneous tissue disorders” (generalised pruritus and urticaria) were also experienced by only 2.1% of patients. It is not surprising that most ADRs were systemic reactions rather than local reactions since i.v. administration results in rapid distribution of the preparation throughout the systemic circulation. The relative frequency of ADRs to i.v. mistletoe delivered alone was more than double the relative frequency of ADRs to i.v. mistletoe delivered prior to chemotherapy. This is probably partly due to ADRs being less likely to be documented for patients who received i.v. mistletoe prior to chemotherapy, since adverse events might have been attributed to chemotherapy instead. Furthermore, with 63% of patients receiving anti-inflammatory corticosteroids (e.g., dexamethasone, prednisolone) as antiemetic or antiallergic medication at least once after i.v. mistletoe infusion and before infusion of chemotherapeutic drugs, it is likely that ADRs to mistletoe, especially pyrexia, were prevented. We therefore believe that the relative frequency of 4.3% (mistletoe alone) of patients experiencing an ADR to i.v. mistletoe provides a more reliable picture than that of 1.6% (mistletoe and chemotherapy).

Despite the fact that increased dose is commonly considered a major risk factor for experiencing an ADR, an obvious relationship between dose and the frequency of ADRs was not observed in the present study. Only Iscador had a higher median ADR dose compared to the median exposure dose. It is possible that an effect of dose on the risk of patients having an ADR is not clear due to their being too few events for a concise investigation. Nevertheless, an interesting observation is that almost all ADRs occurred in response to a first ever exposure to mistletoe, a first exposure to i.v. administration of mistletoe, or to an abrupt increase in dose. Out of 15 patients that continued i.v. mistletoe applications after an ADR, only three experienced subsequent ADRs, suggesting that ADRs were generally one-off events related to first exposure or dose elevation.

Although it is difficult to draw strong conclusions due to large variations in exposure frequencies of different preparation types, Iscador Mali special, Abnoba viscum Quercus, Iscador Mali, and Iscador Quercus resulted in the highest relative frequencies of ADRs. It has been reported previously that Iscador preparations frequently induce fever [6, 37]. Our results coincide with this observation, since 4 of the 6 ADRs (66.7%) to Iscador preparations were fever reactions (pyrexia). This could be related to the manufacturing process of fermentation, unique to Iscador preparations [6, 37, 38]. In contrast, less frequent but more varied ADRs were observed in response to Abnoba viscum Fraxini and Helixor Mali, including skin and hypersensitivity reactions.

Preliminary results of a qualitative study on physicians applying i.v. mistletoe suggests that the safety of i.v. application of mistletoe preparations strongly depends on the speed of infusion (Gunver Kienle, personal communication). Unfortunately no information was available to assess the relationship between infusion speed and ADR risk in the current study.

In the interest of determining whether “off-label” i.v. administration of mistletoe is as safe as the more common s.c. form of administration, which has marketing authorisation, we compared the present i.v. ADR data with results of a previous study on s.c. mistletoe therapy [27]. Although we previously reported on expected effects of s.c. mistletoe applications in addition to ADRs, we decided not to assess expected effects of i.v. mistletoe infusions for the following reasons: (i) expected effects are less defined for i.v. applications since it is an off-label use of mistletoe, (ii) only ADRs are critical for an analysis of safety, while expected effects might be more important for an analysis of efficacy, and (iii) data regarding expected effects is much more vulnerable to reporting bias (e.g., slight rises in body temperature might not be documented as thoroughly as temperatures >38°C). In regard to ADRs, it was found that patients were almost twice as likely to experience an ADR to s.c. therapy compared to i.v. therapy. Also less ADRs were reported per person in response to i.v. therapy and severe ADRs occurred in response to s.c. but not to i.v. therapy. Whether the lower frequency of ADRs in i.v. patients can be partly explained by differing demography and morbidity is unclear. There was no significant difference in age between the groups, but there was a lower percentage of females in the i.v. group and a greater percentage of late stage cancer patients. Female gender has previously been identified as a risk factor for experiencing an ADR, while late stage cancer is associated with a suppressed immune system and might have contributed to a lower frequency of ADRs in i.v. compared to s.c. patients [27, 39–41]. The most notable difference between the ADRs that occurred in response to s.c. applications compared to i.v. applications is a much higher frequency of local reactions and pyrexia after s.c. applications and of skin disorders, such as pruritus, after i.v. applications. This makes sense when considering the pharmacokinetics of each of these administration types. I.v. administration involves immediate delivery of a drug into the systemic circulation leading to rapid achievement of the peak drug concentration, known as *C*_max⁡_. On the other hand, s.c. administration of a drug involves slow absorption into the circulation taking longer to achieve *C*_max⁡_, which by comparison is often lower, but more sustained [42]. Therefore, while i.v. application can rapidly reach a high *C*_max⁡_, thereby increasing the risk of systemic, hypersensitivity type reactions (pruritus, urticaria), s.c. application is more likely to induce a local inflammatory response accompanied by cytokine activation of a febrile response [25, 43]. Furthermore, i.v. application not only results in quicker distribution of a drug than s.c. application, but also in a quicker elimination [42, 44]. Studies comparing the half-life of mistletoe lectins and other molecules of comparable size after application by s.c. or i.v. administration have shown that molecules applied i.v. were eliminated up to three times faster than when applied s.c [45–47]. This finding might also help to explain the much higher frequency of pyrexia observed in s.c. patients compared to i.v. patients. Other possible explanations for the difference in ADR frequencies relate to differences in the way i.v. and s.c. mistletoe therapies are delivered. The majority of i.v. patients (70.5%) also received s.c. mistletoe injections. Since patients usually receive mistletoe via the s.c. route earlier in the treatment plan compared to i.v. mistletoe, it is possible that patients who experienced ADRs to s.c. mistletoe were less likely to later receive i.v. mistletoe. Or even if they still received i.v. mistletoe, a tolerance might have developed over time. Furthermore, s.c. injections are often given 2-3 times per week for many months or years, compared to i.v. infusions which are generally given less frequently and for shorter periods of time. Therefore, there is an increased opportunity for patients receiving s.c. applications to suffer an ADR, or for s.c. applications to be wrongly attributed an ADR.

Although there is limited literature available regarding the safety of i.v. mistletoe applications, especially of high doses, our results tend to be in agreement with the few studies that were found. I.v. mistletoe therapy is often offered with the aim to improve quality of life in a palliative setting, but is also used postoperatively across a range of tumour types to prevent recurrence and concurrent to chemotherapy to reduce ADRs [6, 19]. Our results are in agreement with the findings of Wiebelitz and Beer who concluded that “high dose intravenous mistletoe treatment is safe with few and manageable adverse events” [48]. Comparable to our results, the most common ADRs identified in a series of 17 patients with 107 i.v. applications of high dose mistletoe were temperature related (mild to moderate pyrexia) or skin related (generalised exanthema and pruritus) [48]. In contrast, s.c. applications are associated with frequent injection site reactions, along with pyrexia [25]. A retrospective study focusing on therapy with high doses of mistletoe found i.v. administration to be very well tolerated with only occasional light nausea and/or aching limbs with the very first infusion, while pyrexia was reported in <1% of patients [6]. Other reports on safety and tolerability of i.v. mistletoe applications have ranged from no ADRs at all to rare allergic incidents [18, 49, 50].

Limitations of this like most observational studies include the possibility of missing or erroneous data and assessment of a heterogeneous group of patients in terms of demographic, tumour entities, disease stage, varied doses, and frequencies of i.v. mistletoe applications and of concurrent therapies. On the other hand, this study attempted to provide an accurate picture of the clinical use of i.v. mistletoe therapy and of the safety of this use. There was no control group for this study, meaning that ADRs may have been overreported due to our inability to calculate the frequency of disease-related, other therapy-related, or spontaneous adverse events. Furthermore, since nearly half of the patients received intravenous mistletoe infusions alongside chemotherapy at least once, it is possible that some ADRs to chemotherapy might have been wrongly attributed to mistletoe, or vice versa.

## 5. Conclusions

I.v. infusion of mistletoe preparations by experienced clinicians appears to be safe, with an ADR frequency of almost half of that observed for s.c. mistletoe applications. Furthermore, only mild to moderate ADRs occurred and all patients recovered without sequelae. As a result, prospective studies assessing the efficacy of i.v. application of mistletoe preparations are recommended.

## Figures and Tables

**Table 1:** Characteristics of intravenous mistletoe therapy.

	Patients
Application frequency	
Applied once only	92
2–12 applications	266
>12 applications	117
Duration of therapy	
Less than 1 month	313
1–6 months	83
More than 6 months	79
Other applications of mistletoe	
Intravenous only	123
Intravenous + subcutaneous only	293
Intravenous + intratumoural only	9
Intravenous + subcutaneous + intratumoural	42
Intravenous + other applications*	8

*Other applications were 4  × intrapleural, ascites drainage, intraperitoneal, oral, and inhalation.

**Table 2:** Numbers of patients who were exposed to different mistletoe preparations and adverse drug reactions that occurred.

Preparation	Patients	ADRs*	Relative frequency of ADRs (%)^#^	Types of ADRs
Abnoba viscum (n.s.)	2			
Abnoba viscum Abietis	15			
Abnoba viscum Aceris	2			
Abnoba viscum Amygdali	2			
Abnoba viscum Crataegi	1			
Abnoba viscum Fraxini	133	7 (10)	5.3	Local reaction × 2, pruritus × 2, urticaria × 2, pyrexia, myalgia, hypoaesthesia oral, paraesthesia
Abnoba viscum Mali	2			
Abnoba viscum Pini	4			
Abnoba viscum Quercus	7	1 (1)	14.3	Rash
Total Abnoba viscum	**161**	**8 (11)**	**5.0**	
Helixor (n.s.)	5	0		
Helixor Abietis	106	2 (2)	2.8	Urticaria, pruritus
Helixor Mali	187	8 (12)	4.3	Pyrexia × 5, pruritus × 2, fatigue, infusion site inflammation, headache, hypersensitivity × 2
Helixor Pini	51			
Total Helixor	**321**	**10 (15)**	**3.1**	
Iscador (n.s.)	4			
Iscador Mali	8	1 (2)	12.5	Pyrexia × 2
Iscador Mali c. Arg.	1			
Iscador Mali special	8	2 (3)	25.0	Pyrexia, vomiting, chills
Iscador Pini	2			
Iscador Quercus	9	1 (1)	11.1	Pyrexia
Iscador Quercus special	8			
Iscador Ulmi c. Hg	2			
Total Iscador	**40**	**4 (6)**	**10.0**	

Total	475	22	4.6	

*Number of patients to have an ADR followed by the total number of ADRs in brackets.

^#^Percentage of patients exposed to that preparation type who had an ADR.

n.s.: not specified.

**Table 3:** Received therapy combinations and adverse drug reactions to those therapies.

Therapy combinations			Patients	Patients who had an ADR (relative frequency)
Intravenous mistletoe therapy alone			445	19 (4.3)
Intravenous mistletoe therapy	+	chemotherapy*	187	3 (1.6)
		chemotherapy* + immunotherapy	17	0
		surgery	13	0
		bisphosphonates	12	0
		chemotherapy* + radiation therapy	12	0
		immunotherapy	9	0
		hormone therapy	5	0
		radiation therapy	3	0
		bisphosphonates + hormone therapy	2	0
		signal transduction inhibitors	2	0
		chemotherapy* + hormone therapy + signal transduction inhibitors	1	0
		chemotherapy* + signal transduction inhibitors	1	0

*A total of 201 patients (55% of all patients that had chemotherapy and 42% of all i.v. mistletoe patients) received i.v. mistletoe and chemotherapy within close temporal proximity (very often on the same day). Of these patients, 63% also received corticosteroids as prechemotherapy medication (i.e., dexamethasone or prednisolone) at least once.

**Table 4:** Adverse drug reactions attributed to intravenous mistletoe therapy.

System Organ Class	Preferred term	Total patients	Total events	Incidence (%)
Gastrointestinal disorders	Hypoaesthesia oral	1	1	0.2
	Vomiting	1	1	0.2
Gastrointestinal disorders total		**2**	**2**	**0.2**
General disorders and administration site conditions	Pyrexia	8	10	1.7
	Chills	1	1	0.2
	Fatigue	1	1	0.2
	Infusion site inflammation	1	1	0.2
	Local reaction	2	2	0.4
General disorders and administration site conditions total		**10**	**15**	**2.1**
Immune system disorders	Hypersensitivity	1	2	0.2
Immune system disorders total		**1**	**2**	**0.2**
Musculoskeletal and connective tissue disorders	Myalgia	1	1	0.2
Musculoskeletal and connective tissue disorders total		**1**	**1**	**0.2**
Nervous system disorders	Headache	1	1	0.2
	Paraesthesia	1	1	0.2
Nervous system disorders total		**2**	**2**	**0.2**
Skin and subcutaneous tissue disorders	Pruritus	6	6	1.3
	Rash	1	1	0.2
	Urticaria	3	3	0.6
Skin and subcutaneous tissue disorders total		**10**	**10**	**2.1**

Total		22	32	4.6
